# Neuroimaging in cerebral small vessel disease: Update and new concepts

**DOI:** 10.1590/1980-57642016dn11-040002

**Published:** 2017

**Authors:** Mika Shibuya, Claudia da Costa Leite, Leandro Tavares Lucato

**Affiliations:** 1 MD. Instituto de Radiologia, Hospital das Clínicas da Faculdade de Medicina da Universidade de São Paulo (InRad/HC-FMUSP); DASA - Diagnósticos da América. São Paulo SP, Brazil.; 2 MD, PhD. Departamento de Radiologia e Oncologia da Faculdade de Medicina da Universidade de São Paulo; Instituto de Radiologia do Hospital das Clínicas da Faculdade de Medicina da Universidade de São Paulo (InRad/HC-FMUSP); LIM 44-Ressonância Magnética em Neurorradiologia - Faculdade de Medicina da Universidade de São Paulo. São Paulo SP, Brazil.; 3 MD, PhD. Instituto de Radiologia, Hospital das Clínicas da Faculdade de Medicina da Universidade de São Paulo (InRad/HC-FMUSP); Centro de Diagnósticos Brasil (CDB). São Paulo SP, Brazil.

**Keywords:** neuroimaging, magnetic resonance, small vessel disease, neuroimagem, ressonância magnética, doença de pequenos vasos

## Abstract

In recent years, small vessel disease (SVD) has been recognized for its major impact on cognitive impairment in elderly people, where it is often difficult to separate its effects from those of neurodegenerative diseases individually. SVD is a systemic disease, probably related to diffuse endothelial dysfunction, which affects the perforating arterioles, capillaries and venules in the brain. Although often asymptomatic, it is responsible for almost half of all dementia cases and a significant proportion of stroke cases. Imaging features found on magnetic resonance include recent small subcortical infarctions, lacunes of presumed vascular origin, white matter hyperintensity of presumed vascular origin, prominent perivascular spaces and cerebral microbleeds. The recognition of these imaging findings as a spectrum of the same disease caused by endothelial dysfunction of small cerebral vessels can allow an overall analysis of the disease and thus the development of more effective preventive and therapeutic strategies.

## RESUMO.

Nos últimos anos, a doença de pequenos vasos cerebrais (DPV) tem sido reconhecida por seu grande impacto no comprometimento cognitivo em pacientes mais velhos, sendo muitas vezes difícil separar os efeitos dela e das doenças neurodegenerativas individualmente. Trata-se de uma doença sistêmica, provavelmente relacionada com uma disfunção endotelial difusa, que no encéfalo acomete preferencialmente as arteríolas perfurantes, capilares e vênulas. Apesar de muitas vezes ser assintomática, é responsável por quase metade dos casos de demência e por uma parcela importante dos acidentes vasculares encefálicos. Os achados de neuroimagem encontrados na ressonância magnética incluem pequenos infartos subcorticais recentes, lacunas de origem vascular presumida, hipersinal da substância branca de origem vascular presumida, alargamento dos espaços perivasculares e micro-hemorragias. O reconhecimento desses achados de imagem como espectro de uma mesma doença ocasionada pela disfunção endotelial dos pequenos vasos cerebrais possivelmente permitirá uma análise global da doença e com isso o desenvolvimento de estratégias preventivas e terapêuticas mais eficazes.

## INTRODUCTION

Historically, there has always been a dichotomy in Neurology between the study of dementias, especially Alzheimer's disease, and cerebrovascular disease, with a greater focus on large vessel disease (atherosclerosis). Small vessel disease, described in pathological terms as lipohyalinosis and arteriolosclerosis, has, until recently, always received less attention. However, in the last few years, a synergistic interaction between neurodegenerative diseases and cerebral small vessel disease (SVD) has been shown, making it difficult to separate the effects of each of these diseases individually.^1^ SVD is a clinical-cognitive syndrome, commonly related to aging, and associated with certain neuropathological and neuroimaging findings, with preferential involvement of the cerebral perforating arterioles, capillaries and venules, causing damage to white matter and deep cerebral gray matter. It has a wide range of symptoms, ranging from no/little focal neurological deficit (lacune) to global neurological dysfunction and dementia. In the literature, there are several studies showing a direct association between decline in cognitive performance and number of lesions to the subcortical and periventricular cerebral white matter.^2^

Besides accounting for up to 45% of dementias (considering all age groups), SVD has been found to be responsible for 25% of ischemic strokes in the most recent studies, and thus also leads to potentially relevant long-term sequelae for patients.^3^

Pathophysiology. The pathological substrate of SVD is lipohyalinosis, arteriolosclerosis and fibrinoid necrosis; in increasing degrees of severity. Infiltration by plasma components and inflammatory cells is observed, affecting the vascular walls and the perivascular tissues, resulting in damage to these structures.^3^

The classic pathophysiology is that atherosclerosis of the perforating arteries (micro-atheroma, hence the term lipohyalinosis) or arterial hypertension itself causes ischemia due to structural/functional occlusion (related to loss of vascular self-regulation) or arterial narrowing. But this is presumed to be only the final stage of the disease.^3^

It is important to underscore that although there is a certain association between SVD and several factors commonly associated with cerebrovascular disease, there is some discrepancy between control of these risk factors and the progression of SVD, suggesting that other mechanisms are involved. A recent large study has shown that vascular risk factors account for 70% of the variability of atheromatous disease of large vessels, but no more than 2% of the variability of SVD.^4^

It is now believed that diffuse cerebrovascular endothelial dysfunction is the focal point of pathogenesis of SVD, responsible for failure of the blood-brain barrier (BBB).^3^

The cause of this endothelial dysfunction has not yet been fully elucidated. However, BBB permeability is known to increase with age, especially after 60 years.^3^

The possibility of a genetic component explaining SVD due to endothelial dysfunction has emerged from the study of genetic diseases manifesting as SVD, such as the cerebral autosomal dominant arteriopathy with subcortical infarcts and leukoencephalopathy (CADASIL). Studies in these patients show an increase in the cerebrospinal fluid/serum albumin ratio, suggesting BBB dysfunction; models in rats demonstrate that increased barrier permeability precedes the occurrence of tissue damage in these cases.^3^

In addition, there may be associated factors that accelerate this process, such as deposition of b-amyloid protein (explaining the known link between Alzheimer's disease and SVD), inflammation, immune-mediated factors, high blood pressure, and a diet rich in sodium.^3^^,^ 5 This increase in BBB permeability results in extravasation of plasma components in the vessel wall and perivascular tissues, inflammation and loss of vascular autoregulation, leading to smooth muscle injury, luminal reduction and occlusion as the final stage of the disease.^3^

In animal models with hypertensive and stroke-prone mice, a stereotyped sequence of phenomena is observed following BBB dysfunction, manifested by: accumulation of erythrocytes in the vascular lumen, near the vessel wall; followed by migration of plasma cells and proteins into and through the vascular wall; formation of perivascular microhemorrhages; and vascular occlusions leading to ischemia.^6^

Furthermore, particularly in the cases of venules and capillaries, where the walls are very thin, extravasation goes beyond the wall to perivascular tissue, promoting edema and secondary tissue damage manifested by rarefaction/pallor of myelin and demyelination (anatomopathological substrate known for decades of T2-hyperintense foci in the cerebral white matter). Thus, the hypothesis of diffuse cerebrovascular endothelial dysfunction embraces all known manifestations of SVD.

Role of neuroimaging. Neuroimaging should be descriptive, as the findings should be evaluated according to clinical data. Magnetic resonance imaging (MR) is better than computed tomography (CT) in this analysis.^1^

Even MR has only moderate correlation with clinical and *post mortem* diagnosis in large prospective series of elderly patients.^7^ Despite this, MR has a high negative predictive value, therefore a normal study excludes or makes improbable the clinical diagnosis of SVD. The opposite is also true; a MR disclosing a high SVD burden is highly indicative of a significant chance that cognitive impairment is related to at least one component of SVD.

Nomenclature of MR findings in SVD. A major breakthrough in the study of SVD was the development of a consensus nomenclature for the description of disease-related MR findings. MR imaging features that are markers of SVD included: recent small subcortical infarctions, lacunes of presumed vascular origin, white matter hyperintensity of presumed vascular origin (WMHs), prominent perivascular spaces (PVS) and cerebral microbleeds.

**Figure 1:**
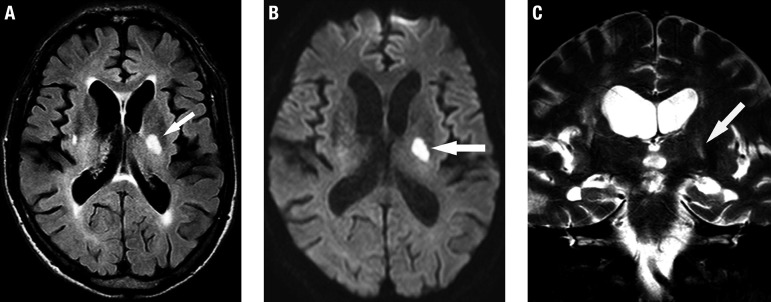
Recent small subcortical infarct. A 75-year-old man with sudden onset of left hemiparesis. Axial FLAIR (A), diffusion-weighted (B) and coronal T2-weighted (C) images demonstrate a recent small infarct in the posterior limb of the left internal capsule (arrows). Note the concomitance of white matter hyperintensities of presumed vascular origin on FLAIR (A) and T2-weighted (C) images. Diffusion-hyperintensity, indicating restriction (arrow in B) corroborates that this represents a recent lesion, and is correlated with the sudden clinical onset. On coronal T2-weighted (C) image, the elongated morphology of the infarction in the superoinferior axis is evident, related to the territory of a perforating artery.

Small subcortical infarcts usually occur in the territory of the perforating arteries (Figure 1). A recent clinical history is possible, and diffusion-weighted imaging (DWI) is fundamental; although in most patients these lesions can appear without apparent symptomatology (silent infarctions). They are usually small in size, generally less than 20 mm across axially and classically located in the territory of perforating arterioles (cerebral white matter, basal ganglia, thalamus and pons). On coronal views, these lesions are sometimes more elongated/cylindrical, delimiting the territory of the occluded arteriole (Figure 1). Recent small subcortical infarcts are hyperintense on DWI, hypointense on an apparent diffusion coefficient (ADC) map, and hyperintense on T2-weighted and fluid-attenuated inversion recovery (FLAIR) images.

Interestingly, a recent subcortical infarction does not always lead to a lacune in long-term follow-up, although this is its most common fate. Alternatively, a recent subcortical infarction may become a nonspecific focus of T2 and FLAIR hyperintensity or even (more rarely) virtually disappear.^3^

The major differential diagnoses for these infarctions are atheromatosis in the ostium of a perforating artery and embolism; however, neuroimaging studies showing multiple markers for SVD increase the possibility of a recent small subcortical infarct.

**Figure 2:**
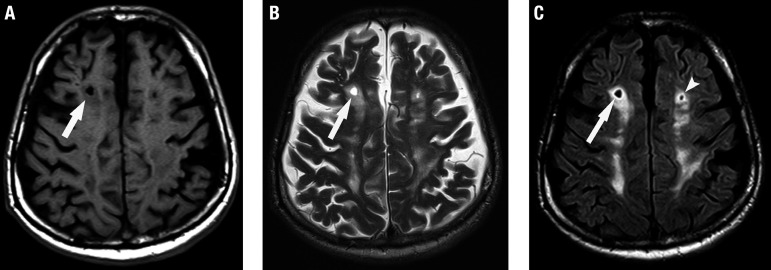
Lacune of presumed vascular origin. A 66-year-old patient with vascular dementia. Axial T1-weighted (A), T2-weighted (B) and FLAIR (C) images show the lacune in the deep white matter of the right centrum semiovale (arrows), with signal intensity similar to CSF in all sequences, with a peripheral rim of gliosis, best seen on FLAIR image (C). Note the concomitance of white matter hyperintensities of presumed vascular origin on FLAIR (C) and T2-weighted (B) images, with corresponding hypointensity on T1-weighted image (A). There is also another lacune in almost the same location, but in the left centrum semiovale (arrowhead in C).

Lacunes of presumed vascular origin (Figure 2) are small cavities located in deep gray and white matter, and also in a territory of perforating artery (white matter, basal ganglia, thalamus and pons). They are typically smaller than 15 mm. If larger, they were probably not caused by SVD. Many lacunes were never symptomatic but appear silently in the brain. They have the same signal intensity as cerebrospinal fluid (CSF) in all sequences. They usually have a peripheral gliotic rim (hyperintense on T2/FLAIR), which may help in the differential diagnosis with other lesions, although this is not a definitive criterion.

**Figure 3:**
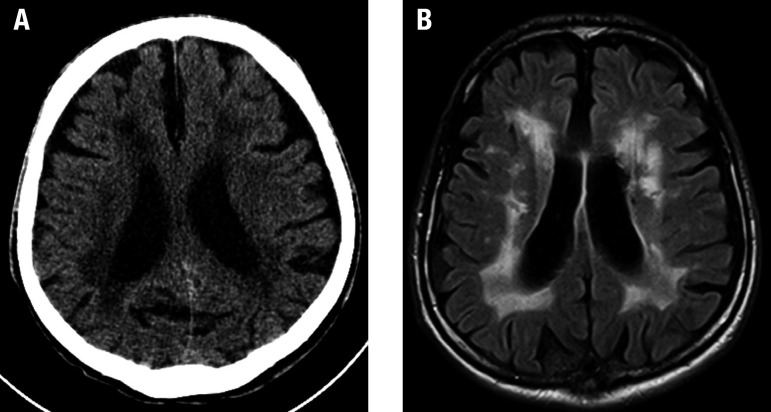
White matter hyperintensities of presumed vascular origin. An 80-year-old woman with cognitive impairment, hypertension and diabetes mellitus. Noncontrast CT image (A) shows diffuse and confluent hypoattenuation of cerebral white matter. Axial FLAIR image (B) demonstrates extensive white matter hyperintensities corresponding with CT hypoattenuation.

White matter hyperintensities of presumed vascular origin are characterized by hyperintense lesions on T2/FLAIR and decreased attenuation on CT (Figure 3). It affects the periventricular/deep cerebral white matter, basal ganglia and pons. These lesions can be relatively symmetric. At least 25% of the cerebral white matter must be affected to be relevant enough to cause symptoms, especially dementia or cognitive impairment.

**Figure 4:**
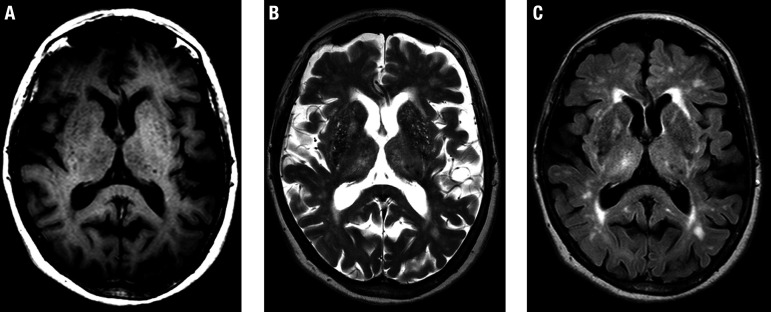
Prominent perivascular spaces. A 75-year-old woman with cognitive decline. Axial T1-weighted (A), T2-weighted (B) and FLAIR (C) images demonstrate multiple prominent perivascular spaces in bilateral basal ganglia regions, smaller than lacunes (see Figure 2), but also with signal intensity similar to CSF in all sequences. There is no T2/FLAIR hyperintense rim. Note the concomitance of white matter hyperintensities of presumed vascular origin on T2-weighted (B) and FLAIR (C) images.

Perivascular spaces, also called Virchow-Robin spaces, are structures covered by the pia mater that surrounds cerebral vessels from the subarachnoid space to the interior of the brain parenchyma. While considered normal in young adults and children, when numerous and prominent in the elderly they are considered another marker of SVD (Figure 4).

The signal intensity is the same as CSF in all sequences, surrounding perforating vessels are more elongated and generally measure less than 3 mm. They are typically located in cerebral white matter and basal ganglia, and do not have a rim of gliosis (in contrast with lacunes). They may have rounded morphology (if observed in a transverse section) or elongated shape (if analyzed along their long axis).

There are two supposed reasons for this association: first, as there is an increase in permeability of the vascular wall, there will be a change in the concentration of proteins in the interstitial fluid that bathes the vessels of the perivascular space, leading to its dilation. Another possible mechanism is that much of the circulation of the interstitial fluid of the perivascular spaces likely occurs through a “milking” mechanism related to habitual dilation of the arteries during systole (pulsatility). This systolic dilatation of the vessel pushes the interstitial fluid through the perivascular space. If the arteriole wall becomes hardened, as in SVD, this “milking” mechanism is lost and leads to an accumulation of interstitial fluid, dilating these perivascular spaces.

**Figure 5:**
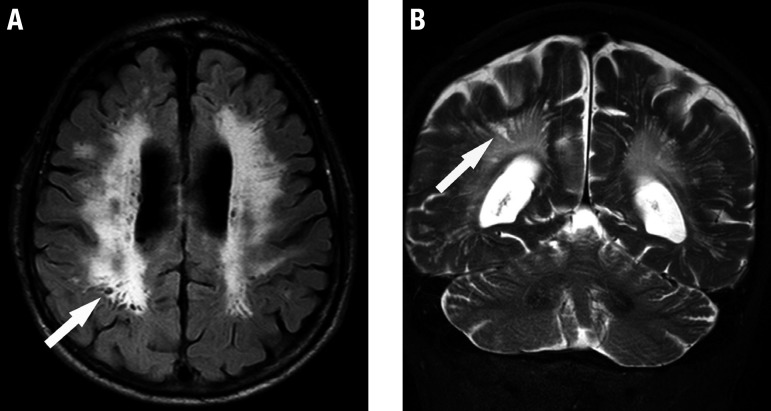
Prominent perivascular spaces. A 75-year-old man with vascular dementia. Axial FLAIR image (A) shows small foci with signal intensity similar to CSF in all sequences, detected in the cerebral white matter, especially in the right parietal lobe. Note one of these foci, which has a rounded morphology (arrow) and is surrounded by T2/FLAIR hyperintensities. This appearance could be more suggestive of a lacune, however, the corresponding coronal T2-weighted image (B) shows that this lesion is elongated (arrow), most probably also corresponding to prominent perivascular spaces. This case illustrates the difficulties usually found in this differentiation.

Although they are extremely common radiological findings and theoretically have typical characteristics, as detailed above, in practice it is difficult to distinguish lacunes, perivascular spaces and white matter hyperintensities of presumed vascular origin (Figure 5). Before classifying these, it is necessary to reformat into other planes and to use volumetric sequences. However, considering that they are part of the same spectrum of SVD, such differentiation lacks clinical significance.

**Figure 6:**
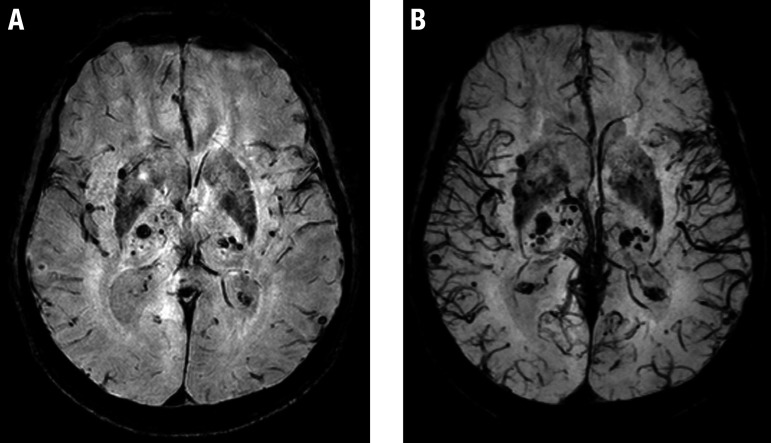
Microbleeds. A 69-year-old man with a history of arterial hypertension and diabetes mellitus. Axial SWI (A) and derived thick minimum intensity projection (mIP) images (B) show multiple hypointense foci scattered throughout the brain, suggestive of microbleeds, predominantly involving the basal ganglia and thalami.

Microbleeds are punctate foci markedly hypointense in sequences that explore magnetic susceptibility phenomena such as T2 gradient-echo (T2*) and especially SWI (susceptibility-weighted imaging). As outlined above, they are also related to diffuse cerebrovascular dysfunction (Figure 6).

Recently, a SVD score that includes lacunes, microbleeds, perivascular spaces and white matter hyperintensities on T2/FLAIR sequences was validated.^8^ This scores ranges from 0 to 4, with 1 point given for each of these characteristics and has potential application as a biomarker of SVD, which can be used in future studies to prevent and treat the disease.

Future directions. Regarding neuroimaging, we can cite two strategies that may have an impact on the understanding and diagnosis of SVD: three-dimensional (3D) FLAIR and double inversion-recovery (DIR) sequences; and also MR permeability studies.

We know that cortical microinfarcts are a relevant risk factor for dementia, detected as small foci restricted to the cortex on autopsies in the elderly population. Microinfarcts are also associated with SVD. It is estimated that the frequency of microinfarcts varies between 16 and 46% in elderly populations not selected according to cause of death.

**Figure 7:**
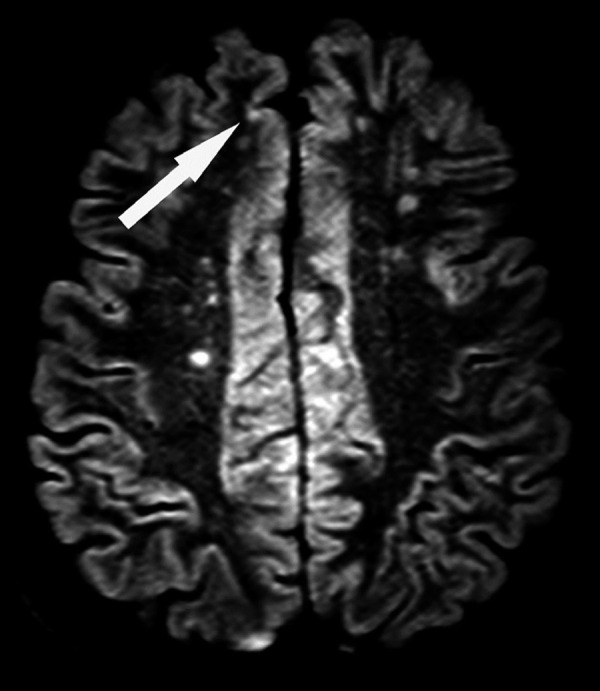
Cortical microinfarct. A 74-year-old man with a history of hypertension and diabetes mellitus. Axial reformatted 3D-DIR image shows a presumed cortical microinfarct, manifesting as a hyperintense lesion (arrow). Note the concomitance of white matter hyperintensities of presumed vascular origin.

This type of lesion, particularly cortical microinfarcts, is practically undetectable on conventional MR studies; however, 3D-FLAIR and DIR sequences can individualize cortical microinfarcts when large^9^ (dimensions above 2-3 mm). This detection is most effective when these sequences are used in higher magnetic fields of at least 3T and above (Figure 7).

**Figure 8:**
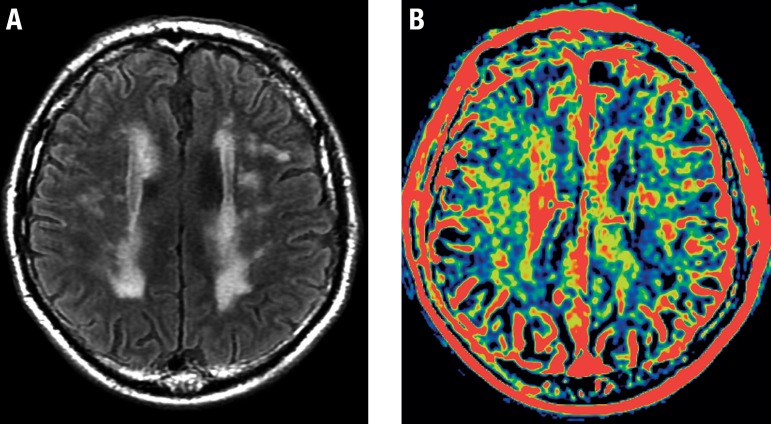
BBB permeability changes in SVD. A 76-year-old male with SVD. Axial FLAIR image (A) shows extensive and confluent white matter hyperintensities of presumed vascular origin. Color map proportional to wash-in rate (B), obtained from a dynamic contrast-enhanced T1 sequence, reveals presumed changes in BBB permeability (in yellow) that show some correspondence with FLAIR hyperintensities.

MR permeability studies have been obtained through the use of gadolinium and by acquiring multiple post-contrast series spanning several minutes. These images are submitted to post-processing, generating color maps that are related to characteristics of BBB permeability. In patients with SVD, these color maps can show some changes that potentially serve as a surrogate marker of endothelial dysfunction (Figure 8).

## CONCLUSIONS

Although the knowledge on SVD is relatively limited, this disease is extremely important, responsible for almost half of all cases of dementia and for many cases of lacunar stroke.

Neuroimaging plays a fundamental role in identifying the characteristic findings of this entity. More important than individualizing and classifying each of these lesions, is recognizing that they are part of the spectrum of the same disease, and an overall analysis of all components of SVD is necessary in MR studies.

It should be emphasized that, in face of the new findings, the recognition of SVD as an endothelial disease associated with glial and neuronal impairment is fundamental for the development of effective preventive and therapeutic strategies.
